# Evaluating the Causal Relationship Between Human Blood Metabolites and the Susceptibility to Alopecia Areata

**DOI:** 10.1111/jocd.70248

**Published:** 2025-05-20

**Authors:** Xiaoli Lei, Yi Qu, Jiaxi Huang

**Affiliations:** ^1^ The First Clinical Medical College Shandong University of Traditional Chinese Medicine Jinan China; ^2^ Department of Pharmacy Huoqiu County First People's Hospital Luan China

**Keywords:** 1400 blood metabolites, alopecia areata, causality, Mendelian randomization

## Abstract

**Background:**

Alopecia areata, a common autoimmune disease, is not fully understood in terms of its cause. However, research suggests that an imbalance in specific blood metabolites may trigger immune system dysfunction, leading to an attack on hair follicles and ultimately resulting in alopecia areata.

**Methods:**

Two‐sample MR analysis was conducted to investigate the causal relationship between plasma metabolites and alopecia areata using various methods. Heterogeneity and pleiotropy were assessed, robustness of findings evaluated, and reverse MR performed for effect analysis.

**Results:**

The MR analysis found a positive causal relationship between alpha‐ketoglutarate, propionylcarnitine (c3) and other metabolites with alopecia areata risk. Conversely, xylose 3‐(3‐hydroxyphenyl)propionate, glycochenodeoxycholate glucuronide (1) along with other metabolites, showed a protective effect against alopecia areata development. Both BWMR and MR‐PRESSO confirmed the accuracy of the above results. Reverse MR revealed no reverse causality between plasma metabolites and AA. The robustness of the results was confirmed using the leave‐one‐out method, which demonstrated no influential instrumental variables affecting the outcomes while accounting for heterogeneity and eliminating horizontal gene pleiotropy effects on estimating causal effects.

**Conclusion:**

This study establishes a causal relationship between plasma metabolism and alopecia areata, enhancing our understanding of its underlying mechanisms. These findings also provide valuable references for future screening and prevention strategies.

## 1 Introduction

Alopecia areata (AA) is a prevalent chronic organ‐specific autoimmune skin disease characterized by focal circular or round patches of hair loss, which can affect various areas such as the scalp, eyebrows, eyelashes, beard, and armpit hair [1]. The estimated global prevalence of AA is 0.10% of the total population, indicating that approximately 8.3 million people worldwide are affected. The estimated prevalence of AA in adults and children is 0.12% and 0.03%, respectively. The highest estimated prevalence is in the Asian region (including Southeast Asia, East Asia, high‐income Asia Pacific, and South Asia), and the lowest is in the African region (including West Sub Saharan Africa, Southern Sub Saharan Africa, Eastern Sub Saharan Africa, and Central Sub Saharan Africa), with a prevalence rate of 0.14% in the East Asian population [2]. Although AA can occur at any age, it most commonly manifests in young individuals between the ages of 30 and 40 [3]. Although the exact cause of AA remains unclear, genetic factors and immune dysregulation have been identified as key contributors to its development [4]. These triggers initiate an autoimmune response involving Th1 type T cells that target and attack the hair follicles. This leads to disruption of immune homeostasis along with extensive infiltration of CD8^+^ and CD4^+^ T cells [5]. Interferon‐gamma secretion is induced through signaling pathways mediated by Janus kinase (JAK)‐1 and JAK2 within the epithelium surrounding hair follicles. Furthermore, stimulation of IL‐2, IL‐15, and other cytokines occurs alongside inflammation‐hair follicle interactions contributing to AA in affected patients [6]. Traditional treatment for AA includes glucocorticoids, minoxidil, contact immunotherapy, and immunosuppressants such as methotrexate and cyclosporine [7]. Glucocorticoids are the primary drugs used to treat AA due to their significant short‐term effects. However, they have drawbacks such as prolonged treatment duration and high recurrence rates, which can severely impact patients' mental health and quality of life. Recently, new treatments for AA have emerged including oral JAK inhibitors, platelet‐rich plasma therapy, narrow spectrum UVB phototherapy, and low energy laser therapy [8]. Nevertheless, these treatment methods either come with a high cost or inconvenience that hinders patient adherence. Furthermore, their efficacy and safety still require further evaluation.

The presence of plasma metabolites can exert an influence on disease susceptibility and serve as practical markers for personalized medicine and disease monitoring [9, 10]. Comprehending the causal role of metabolites in disease etiology can offer easily controllable intervention points for treatment [11]. In the context of plasma metabolite genome‐wide association studies (GWAS), novel gene‐metabolite associations have been identified and utilized to elucidate potential targets for various traits and diseases. These findings may contribute to our understanding of genetic regulation in human metabolism, facilitate future meta‐analyses of prospective programs, and provide a valuable resource for identifying targets suitable for behavioral and pharmacological interventions [12]. Plasma metabolites influence AA progression through several key biological pathways supported by existing mechanistic and clinical studies. The JAK–STAT signaling pathway is central, with interferon‐γ and γc cytokines (e.g., IL‐2, IL‐7, IL‐15) activating JAK–STAT cascades, driving CD8^+^ T‐cell infiltration into hair follicles and disrupting immune privilege [13]. This pathway is clinically validated by the efficacy of JAK inhibitors like baricitinib [14]. Leptin, a pro‐inflammatory mediator, induces the production of pro‐inflammatory cytokines (e.g., TNF‐α and IL‐6) and reactive oxygen species in cultured monocytes. It also stimulates macrophages to generate chemokines and shifts the Th1/Th2 balance toward a Th1 phenotype [15]. Although leptin is hypothesized to promote Th1 polarization via JAK–STAT signaling, clinical studies show no significant differences in serum leptin levels between AA patients and controls, suggesting tissue‐specific actions rather than systemic effects [16]. Vitamin D plays a crucial role in modulating both innate and adaptive immune responses, and its deficiency has been linked to increased susceptibility to autoimmune disorders and infections [17]. In AA, multiple studies have reported reduced serum vitamin D levels in patients [18]. Emerging biomarkers such as lipocalin‐2 and insulin are under investigation for their roles in AA pathogenesis, potentially linking metabolic disturbances to immune activation [19].

Genetic variation can influence metabolite levels by modulating the expression of enzyme‐encoding genes, altering enzyme structures, or completely inactivating enzymes due to protein‐truncating variants [20]. For instance, HLA‐II gene variants impact antigen presentation efficiency [21], and rare variants in KRT82 result in the release of keratin degradation products [22]. These alterations may perturb metabolite levels, thereby disrupting specific metabolic pathways such as lipid metabolism dysregulation or oxidative stress toxin accumulation. Such metabolic disturbances can elevate the risk of AA either through immune dysregulation involving abnormal activation of CD8^+^ T cells, CD4^+^ T cells, and NK cells [23] or by directly damaging the hair follicle microenvironment, as exemplified by maleic acid‐induced disruption of cellular redox balance [24]. As intermediate phenotypes linking genes and phenotypes, metabolites elucidate the mechanisms underlying susceptibility genes (e.g., IL13 and KIAA0350) identified in GWAS of AA [25].

Mendelian randomization (MR) employs genetic variation as a robust tool, closely linked to modifiable risk factors, for investigating causal effects on outcomes [26]. Observational studies are prone to potential confounding factors and reverse causality, which can introduce bias in the results, while MR studies utilize single nucleotide polymorphisms (SNPs) that are closely associated with potential exposure factors and do not vary with lifestyle or socioeconomic factors that may confound traditional observational studies, analyzing the causal relationship between exposure factors and outcomes at the genetic level [27, 28]. The fundamental advantage of the MR method lies in its ability to mitigate bias caused by confounding factors and reverse causality [29, 30]. To ensure the credibility of MR research results, genetic variation needs to meet three key assumptions: (1) genetic variation is related to exposure factors; (2) genetic variation is not related to confounding factors; (3) genetic variation only affects the occurrence of outcomes through exposure factors [31]. With the aim of determining effective interventions for promoting health benefits [32], this study sought to explore the relationship between 1400 plasma metabolites and AA using bidirectional two‐sample Mendelian randomization analysis, thereby providing additional evidence for the prevention and treatment of AA.

## 2 Materials and Methods

### 2.1 Design

**FIGURE 1:**
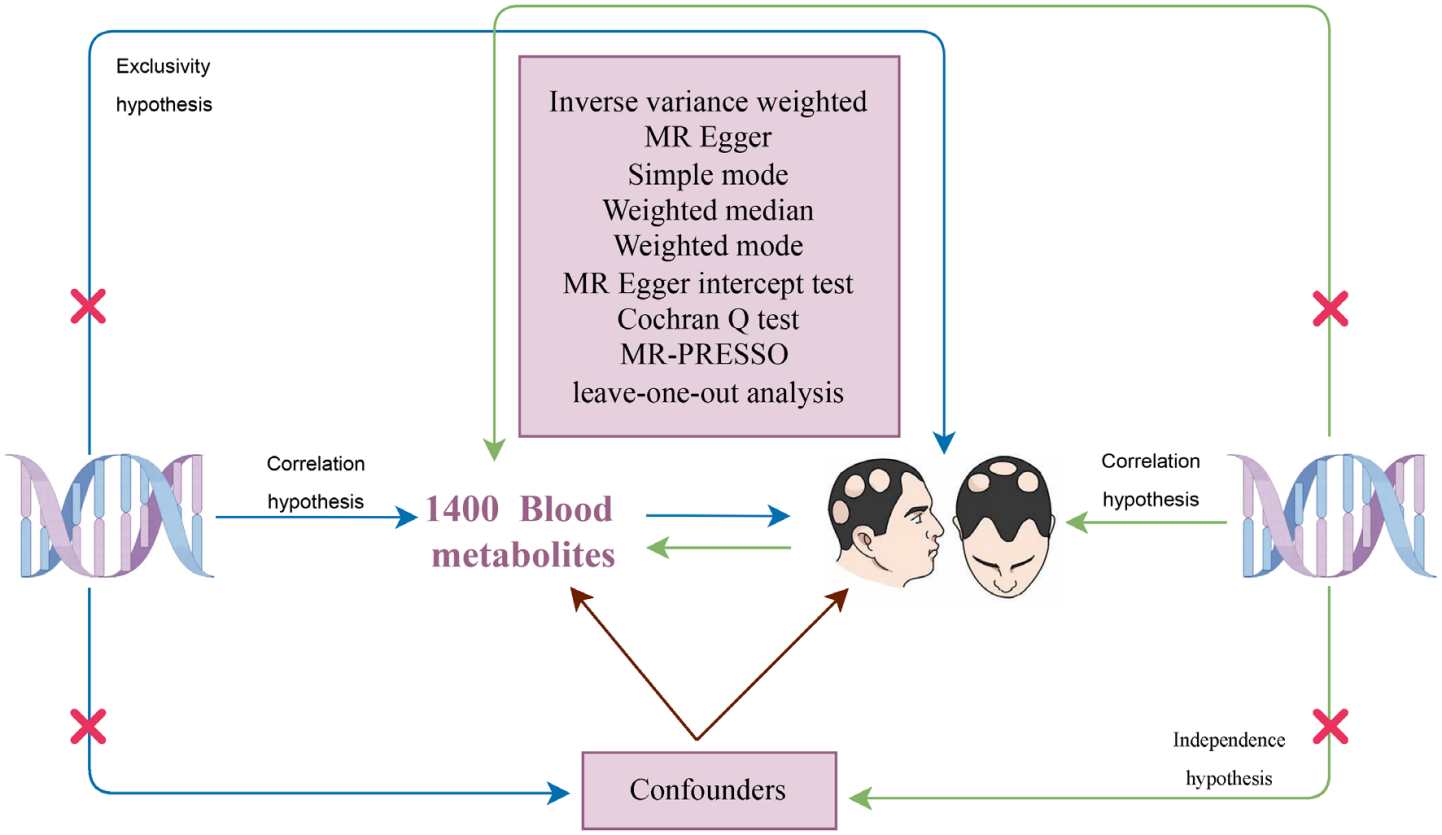
A two‐way MR design.

A two‐sample two‐way MR analysis was employed to evaluate the relationship between plasma metabolites and AA. GWAS data were collected for 1400 plasma metabolites and AA. To ensure reliable results, three fundamental assumptions of MR needed to be met as follows: (1) The instrumental variable SNPs included in the final analysis should exhibit strong associations with the exposure variables (1400 plasma metabolites or AA); (2) SNPs must be independent of known confounders that affect 1400 plasma metabolites or AA; (3) SNPs should only influence the outcome variables (AA or 1400 plasma metabolites) through the exposure variables (1400 plasma metabolites or AA) (see Figure 1).

### 2.2 Data Source

The study encompassed a total of 1400 plasma metabolites, which were classified into four distinct populations: 8299 cases from Europe, 108 cases from South Asia, 104 cases from East Asia, and 60 cases from Africa. These datasets comprise approximately 15.4 million SNPs and can be accessed through the GWAS database query (https://www.ebi.ac.uk/gwas/). Specifically, the GWAS data for the European population can be found under access numbers GCST90199621‐90201020, whereas non‐European GWAS data are accessible via access numbers GCST90201021‐902040632 [12]. The Finngen database (https://finngen.gitbook.io/documentation/) contributed to this research by providing medical records of 767 AA cases and a control group consisting of 394 105 individuals. All participants in this dataset share European ancestry, with a total of 19 682 487 SNPs available for analysis. It is important to note that all aggregated data used in this article are publicly available and can be downloaded free of charge. Furthermore, each GWAS study mentioned in the paper has obtained ethical approval from the appropriate regulatory agency.

### 2.3 Tool Variable Selection

The instrumental variables (IVs) were selected as SNPs that showed a close association with 1400 plasma metabolites (*p* < 1.0 × 10^−5^). Linkage disequilibrium (LD) analysis was performed using European genome sample data, with the operating parameters set at *r* ^2^ < 0.001 and kb = 10 000. The strength of the instrumental variables was evaluated by calculating the *F*‐statistic value. An *F* > 10 indicates no weak instrumental variable bias, whereas instrumental variables with *F* < 10 are eliminated. The *F* value is calculated as follows: *F* = [(*n* − *k* − 1)/*k*] *r* ^2^/(1 − *r* ^2^), where *r* ^2^ represents the exposure variance explained by SNPs, *n* is the number of samples in the exposure data, and *k* is the number of SNPs used. The computation formula for *r* ^2^ is given by: *r* ^2^ = 2 × beta^2^ × EAF × (1 − EAF)/[beta^2^ × (1 − EAF) + SE^2^ × *N* × 2EAF × (1 − EAF)]. Here, EAF denotes effect allele frequency, *β* represents allele effect value, and SE stands for standard error. A harmonization process was also conducted to remove palindromic and ambiguous SNPs with minor allele frequency (MAF > 0.01), ensuring that alleles do not influence the results. The SNPs for the exposure factor were identified via the GWAS Catalog (https://www.ebi.ac.uk/gwas/), with confirmation of no confounding SNPs.

### 2.4 Analysis Methods

#### 2.4.1 MR Analysis

The inverse variance weighted (IVW) method assumes that all SNPs are valid instrumental variables, and calculates the weighted average of effect sizes to determine the degree of correlation between exposure and outcome. The results obtained from the IVW random effects model remain robust even when there is heterogeneity in instrumental variable selection, making the parameter estimation more conservative and realistic [33].

Therefore, the outcomes derived from the IVW random effects model are considered as the primary result in Mendelian randomization analysis. Additionally, the MR‐Egger regression method provides a relatively stable estimate of effect [34]. The weighted median (WME) method combines data from multiple genetic variants into a single causal estimate [35]. Weighted mode (WM) and simple mode (SM) are model‐based estimation approaches that aggregate SNPs with similar causal effects and provide causal effect estimates for most clustered SNPs. These methods serve as supplementary tools in MR analysis to assess the reliability and stability of outcomes.

#### 2.4.2 Sensitivity and Pleiotropy Analysis

Cochran's *Q* test was utilized for heterogeneity analysis. If *p* < 0.05, indicating the presence of heterogeneity, the random effects model was employed to estimate causal effects; otherwise, the fixed effects model was applied. The MR‐Egger regression test was adopted to assess horizontal pleiotropic effects, with the estimated effect value represented by the intercept [36]. To identify and eliminate horizontal pleiotropic outliers, we employed the Mendelian randomized pleiotropy residual and outlier (MR‐PRESSO) test [37]. Sensitivity analysis was conducted using a leave‐one‐out method to examine the influence of each SNP on the results.

#### 2.4.3 BWMR Analysis

Conventional MR Test methods may not be able to clear the uncertainty of weak level pleiotropy and weak effect, thus causing the causal relationship between exposure and outcome. Bayesian weighted Mendelian randomization (BWMR), based on causal inference, can explain the uncertainty of weak effect and weak level pleiotropy effect, and better prove the causal relationship between exposure and outcome [38]. In this study, the BWMR method fully verified the causal association between plasma metabolites and AA.

### 2.5 Statistical Methods

Version 4.3.2 of the R software, along with the “TwoSampleMR,” “MRPRESSO” package, was utilized for conducting statistical analyses, and the RadialMR package was used to remove abnormal SNPS. The findings were reported as odds ratios (OR) accompanied by their respective 95% confidence intervals (CI). All tests adhered to a significance level of *α* = 0.05. The *p* value is corrected by FDR. Furthermore, a reverse MR analysis was carried out to explore possible inverse causal associations between the 1400 plasma metabolites identified in the initial MR analysis and AA. The Steiger test was also used to infer the direction of the causal relationship between exposure and outcome.

## 3 Results

### 3.1 Positive MR Analysis Results

**FIGURE 2:**
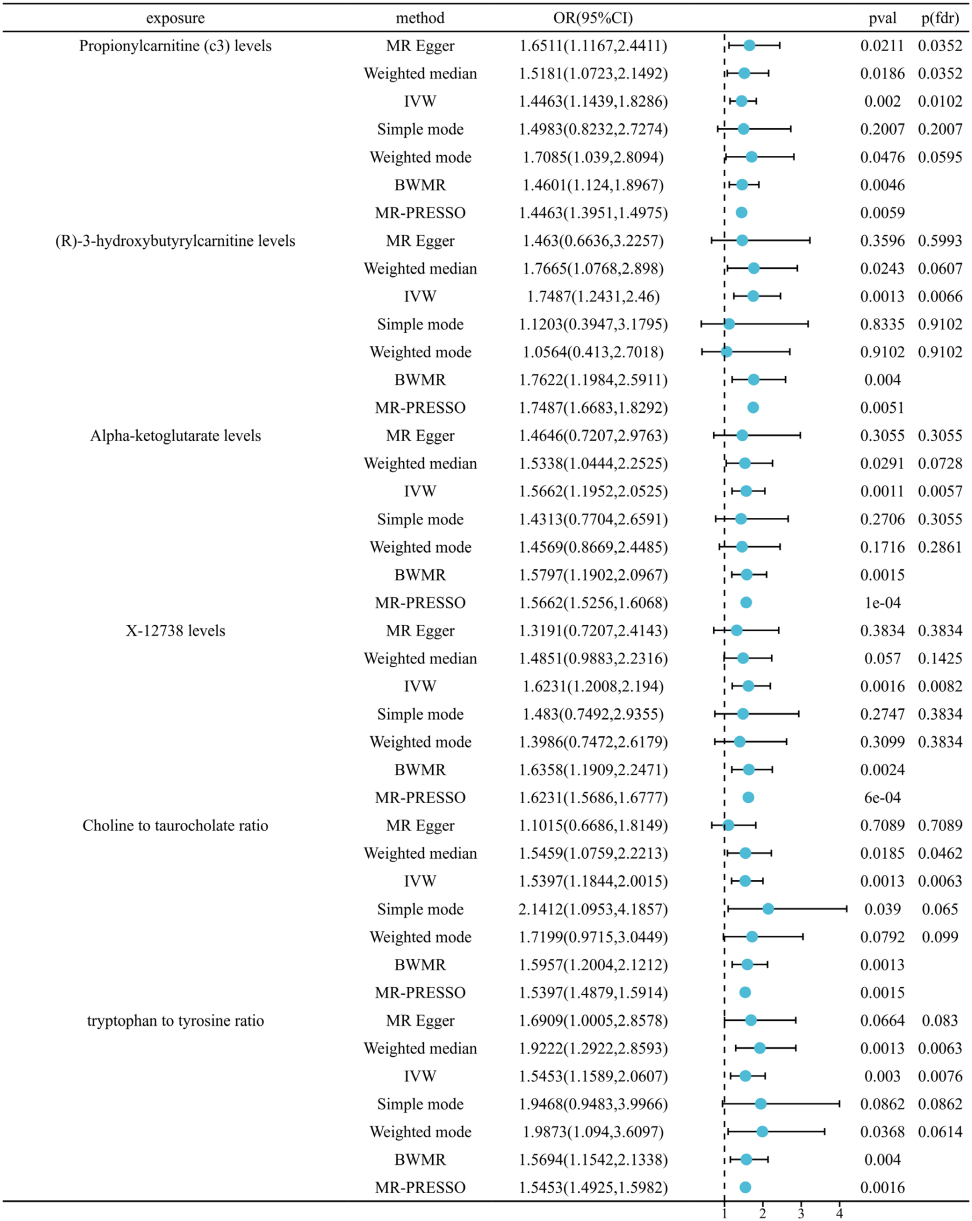
Positive causal relationship between 1400 plasma metabolites and AA based on IVW and BWMR.

**FIGURE 3:**
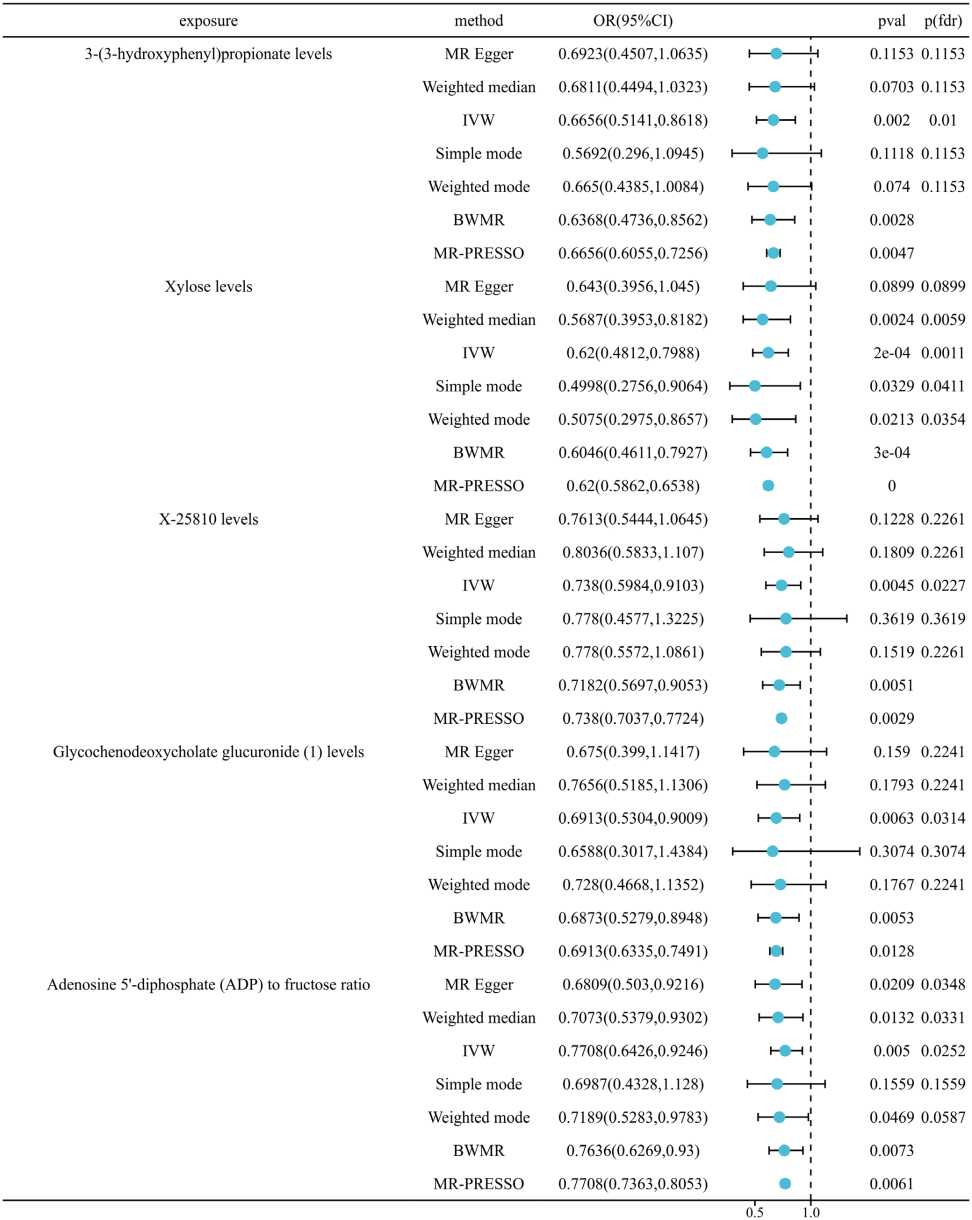
Negative causal relationship between 1400 plasma metabolites and AA based on IVW and BWMR.

Using 1400 plasma metabolites as exposure factors and AA as the outcome variable, we applied a *p* < 1.0 × 10^−5^ for screening purposes. Palindromes or incompatible alleles were excluded, resulting in a total of 27 264 SNPs obtained from 1294 plasma metabolites. Furthermore, all included SNPs exhibited an *F* statistic > 10, indicating that our study is robust against weak instrumental variable bias. The relevant findings are presented in Figures 2 and 3.

#### 3.1.1 May Be Positively Related to AA

IVW shows a positive correlation with the appearance of AA Propionylcarnitine (c3) levels (OR = 1.4462, 95% CI: 1.14385–1.8286, *p*(fdr) = 0.0102), (R)‐3‐hydroxybutyrylcarnitine levels (OR = 1.4601, 95% CI: 1.1240–1.8967, *p*(fdr) = 0.0066), alpha‐ketoglutarate levels (OR = 1.5662, 95% CI: 1.1952–2.0525, *p*(fdr) = 0.0057), X‐12738 levels (OR = 1.6231, 95% CI: 1.2008–2.1940, *p*(fdr) = 0.0082), choline to taurocholate ratio (OR = 1.5397, 95% CI = 1.1844–2.0015, *p*(fdr) = 0.0063), tryptophan to tyrosine ratio (OR = 1.5453, 95% CI = 1.1589–2.0607, *p*(fdr) = 0.0076). BWMR shows a positive correlation with the appearance of AA propionylcarnitine (c3) levels (OR = 1.4601, 95% CI = 1.1240–1.8967, *p* = 0.0046), (R)‐3‐hydroxybutyrylcarnitine levels (OR = 1.7622, 95% CI = 1.1984–2.5911, *p* = 0.0040), alpha‐ketoglutarate levels (OR = 1.5797, 95% CI = 1.1902–2.0967, *p* = 0.0015), X‐12738 levels (OR = 1.6358, 95% CI = 1.1909–2.2471, *p* = 0.0024), choline to taurocholate ratio (OR = 1.5957, 95% CI = 1.2004–2.1212, *p* = 0.0013), tryptophan to tyrosine ratio (OR = 1.5694, 95% CI = 1.1542–2.1338, *p* = 0.0040). In addition, MR‐PRESSO analysis also confirmed that these six plasma metabolites were positively correlated with AA (Figure 2).

The sensitivity *Q* test results revealed no significant heterogeneity among the selected instrumental variables. Furthermore, the MR‐Egger analysis and MR‐PRESSO Global Test demonstrated that there was no observed pleiotropy in the levels of plasma metabolites examined in this study (Appendix S1). Additionally, employing the leave‐one‐out method for analysis did not identify any significant outliers, thus confirming the robustness of our MR Study findings (Appendix S1).

#### 3.1.2 May Be Negatively Related to AA

IVW showed a negative correlation with AA 3‐(3‐hydroxyphenyl)propionate levels (OR = 0.6656, 95% CI = 0.5141–0.8618, *p*(fdr) = 0.0010), xylose levels (OR = 0.62, 95% CI = 0.4812–0.7988, *p*(fdr) = 0.0011), X‐25810 levels (OR = 0.738, 95% CI = 0.5984–0.9103, *p*(fdr) = 0.0227), glycochenodeoxycholate glucuronide (1) levels (OR = 0.6913, 95% CI = 0.5304–0.9009, *p*(fdr) = 0.0314), adenosine 5′‐diphosphate (ADP) to fructose ratio (OR = 0.7708, 95% CI = 0.6426–0.9246, *p*(fdr) = 0.0252). BWMR shows a positive correlation with the appearance of AA 3‐(3‐hydroxyphenyl)propionate levels (OR = 0.6368, 95% CI = 0.4736–0.8562, *p* = 0.0028), xylose levels (OR = 0.6046, 95% CI = 0.4611–0.7927, *p* = 0.0003), X‐25810 levels (OR = 0.7182, 95% CI = 0.5697–0.9053, *p* = 0.0051), glycochenodeoxycholate glucuronide (1) levels (OR = 0.6873, 95% CI = 0.5279–0.8948, *p* = 0.0053), ADP to fructose ratio (OR = 0.7636, 95% CI = 0.6269–0.93, *p* = 0.0073). In addition, MR‐PRESSO analysis also confirmed that these five plasma metabolites were negatively correlated with AA (Figure 3).

The Sensitivity *Q* test results revealed no significant heterogeneity among the selected instrumental variables. Furthermore, the MR‐Egger analysis and MR‐PRESSO Global Test demonstrated that there was also no observed pleiotropy in the levels of plasma metabolites examined in this study (Appendix S1). No noteworthy outliers were detected in the leave‐one‐out method analysis results, thereby bolstering the credibility of our MR research findings (Appendix S1).

### 3.2 Reverse MR Analysis

Positive plasma metabolites were used as the outcome of reverse MR, and AA was used as the exposure factor to investigate whether there was reverse causality between plasma metabolites and AA. The results reveal the relationship between AA and propionylcarnitine (c3), 3‐(3‐hydroxyphenyl)propionate, (R)‐3‐hydroxybutyrylcarnitine, xylose, alpha‐ketoglutarate, X‐12738, X‐25810, glycochenodeoxycholate glucuronide (1), ADP to fructose ratio, choline to taurocholate ratio, and tryptophan to tyrosine ratio showed no causal correlation between these 11 plasma metabolites, and the Steiger test also confirmed that plasma metabolites had a positive causal correlation with AA (Appendix S1).

## 4 Discussion

AA is a prevalent nonscarring form of hair loss that affects individuals across all generations and can be challenging in severe and recurrent cases [39]. Relevant studies have demonstrated the systemic nature of AA, with integrated scalp and serum biomarker models providing unique insights into the phenotype of moderate to severe AA, emphasizing the necessity for systemic treatment [40]. These studies underscore the systemic character of AA as a disease and emphasize the significance of blood metabolites in comprehending its pathogenesis and developing novel therapies [40]. Plasma metabolites serve as crucial biochemical indicators reflecting various physiological and pathological processes within the human body. By conducting GWAS database analysis to identify highly correlated or specifically associated components among 1400 plasma metabolites, followed by validating their regulation by genes, it can be inferred that these components may influence the onset and progression of AA. Exploring the relationship between 1400 blood metabolites and AA could provide a foundation for future deeper and more accurate diagnosis as well as treatment strategies. Furthermore, designing intervention or drug treatment programs targeting these components holds promise for noninvasive diagnosis and targeted therapy for AA. This systematic feature indicates that the pathogenesis of AA is likely to be closely associated with the imbalance of metabolic networks regulated by genetic factors.

Genes regulate plasma metabolite levels by encoding metabolic enzymes [41], transporters [42], and regulatory factors [43]. Changes in enzyme activity or transporter expression can reshape the plasma metabolite profile. Epigenetic modifications, such as DNA methylation and histone modification, further influence carbohydrate, lipid, and amino acid metabolite concentrations, thereby disrupting immune‐metabolic pathways. IL2RA gene variation disrupts Treg cell homeostasis by interfering with IL‐2 signaling, leading to immune imbalance [44, 45]. IL23R polymorphism enhances Th17 cell‐mediated inflammatory responses [46]. Abnormal cholesterol metabolic intermediates impair hair follicle growth and immune regulation via JAK–STAT and PPAR pathways [47]. CXCL10 and other chemokines exhibit abnormal secretion, intensifying CD8^+^ T cell and Th1 cell infiltration and forming an inflammatory amplification loop [48]. Gene‐driven metabolic disorders indirectly exacerbate the pathological process of AA through oxidative stress imbalance, DNA synthesis defects, and microbiota‐host interactions [49]. GWAS have confirmed that AA‐related gene variations co‐localize with changes in plasma metabolite levels and disease risk loci [50, 51]. Genetic factors influence the immunoinflammatory microenvironment through metabolic reprogramming, ultimately resulting in the loss of hair follicle immune privilege and abnormal hair cycle regulation [52, 53]. In this study, a two‐sample MR method was employed to comprehensively investigate the potential causal association between 1400 plasma metabolites and AA. To ensure the accuracy and reliability of our findings, we excluded common genetic variants that may cause confounding bias in epidemiological investigations and only selected SNPs with strong associations with the variables under investigation. Moreover, the large sample size of our MR analysis provided robust correlation evidence, which strengthened the persuasiveness of our research conclusions. We excluded pleiotropic genes, heterogeneity, and outliers from our analyses while performing sensitivity analyses to ensure result robustness. Our Sensitivity *Q* test indicates that there is no heterogeneity, indicating that the selected instrumental variable SNP is not affected by environment, personal differences, and other factors, and will not bring bias to the research results. The MR‐Egger analysis reveals that 11 plasma metabolites do not have horizontal pleiotropy, and when there is horizontal pleiotropy, it violates the exclusivity hypothesis of MR And does not exclude the influence of confounders on the study results, thus drawing a wrong causal relationship. The results of the Steiger test and BWMR analysis both suggested that SNP could affect outcome changes only through exposure factors, and these analysis methods also suggested that there was a strong correlation between SNP and exposure factors, and excluded independent confounders, which brought the possibility of false positives to the study results and further increased the robustness of the results. Under the assumption that all IVs were valid [54], we used IVW as our primary means of analysis to establish a causal relationship between metabolites and AA. In addition, both MR‐PRESSO and BWMR assays confirmed a causal association between plasma metabolites and AA. Our results revealed significant associations between 11 metabolites and AA; however, chemical properties for five metabolites remain unknown. Alpha‐ketoglutarate, propionylcarnitine (c3), (R)‐3‐hydroxybutyrylcarnitine, choline to taurocholate ratio, tryptophan to tyrosine ratio, X‐12738 were positively causally associated with AA, while xylose and 3‐(3‐hydroxyphenyl) propionate, X‐25810, glycochenodeoxycholate glucuronide (1), ADP to fructose ratio among other metabolites acted as protective factors against it. AA was treated as exposure while plasma metabolites served as outcomes in this study's reverse MR analyses which showed a negative causal association with one plasma metabolite but positive ones for two others.

Alpha‐ketoglutarate levels ( αKG) serve as the pivotal link between carbon and nitrogen metabolism. αKG, a crucial metabolite in the tricarboxylic acid (TCA) cycle [55], exerts its influence through diverse mechanisms including mTOR and ATP synthase inhibition, regulation of DNA and histone demethylation [56]. Histone methylation plays a central role in governing chromatin structure, gene activity, and other chromatin‐related biological processes. Notably, histone demethylation significantly impacts the field of epigenetics. By modulating histone methylation modifications, αKG may potentially regulate the expression of an array of environmental response genes and thereby participate in the pathogenesis regulation of AA; however, further investigation is warranted to elucidate the specific relationship between αKG and AA. Propionylcarnitine (C3) serves as a crucial diagnostic marker for congenital fatty acid oxidation disorders and an important indicator for physiological processes such as energy metabolism, mitochondrial and peroxisome β‐oxidation activity defects, insulin resistance, and metabolic disorders [57]. Given the higher prevalence of metabolic disorders in patients with AA [58], it suggests a potential positive correlation between propionylcarnitine (C3) and AA. Therefore, there is evidence to support a positive association between propionylcarnitine (C3) and AA. (R)‐3‐hydroxybutyrylcarnitine is currently found to be a potential biomarker for febrile seizure in children but has not been reported in other diseases [59]. This study validated (R)‐3‐hydroxybutyrylcarnitine as a potential marker of AA at the genetic level using MR Methods. Tryptophan and tyrosine are aromatic amino acids, which are essential amino acids in the human body and participate in many important biological processes in the human body, such as oxidative stress, regulation of immunity, and metabolism. Studies have shown that tyrosine and tryptophan are closely related to metabolic syndrome, and significantly increased plasma tyrosine and tryptophan levels have been found in Chinese patients with metabolic syndrome, thus affecting glucose and lipid metabolism and increasing the risk of heart disease, diabetes, stroke, and chronic neurodegenerative diseases [60, 61]. AA is an autoimmune system disease; current studies have revealed that AA is closely associated with vitiligo, type 1 diabetes, lupus erythematosus, autoimmune thyroid disease, Addison's disease, and insulin resistance, and AA can also increase the risk of long‐term metabolic syndrome. In addition, recent studies have shown that AA is also associated with cardiovascular risk factors (such as obesity, insulin resistance, dyslipidemia) and vascular endothelial dysfunction, thereby increasing the risk of cardiovascular disease in patients with AA [62]. However, the tryptophan to tyrosine ratio has not yet been reported. Therefore, we recommend that the tryptophan to tyrosine ratio may affect the disease progression of AA by influencing the metabolic process of the body.

Xylose, as a functional monosaccharide, effectively stimulates bifidobacteria proliferation in the intestine [63], enhances body immunity, inhibits α‐glucosidase and α‐amylase activities while reducing blood sugar levels [64]. Metabolic abnormalities and pathological states induced by hyperglycemia are characterized by increased oxidative stress (OS), inflammatory response formation leading to IR formation and fat deposition [65]. Elevated OS levels trigger the release of pro‐inflammatory cytokines. AA is closely associated with OS and autoimmune diseases primarily through inducing inflammation, inducing apoptosis promotion, and reduction in immune tolerance. 3‐(3‐Hydroxyphenyl)propionate is one of the phenylpropanoids from lignin, often used as a sole carbon and energy source for a subset of bacteria [66]. Some researchers analyzed the efficacy and adverse drug reactions of cisplatin combined with gemcitabine chemotherapy in patients with lung adenocarcinoma using metabolomics methods and found that 3‐(3‐hydroxyphenyl)propionate is a potential biomarker of blood toxicity in patients with lung adenocarcinoma [67]. We demonstrated a significant causal relationship between 3‐(3‐hydroxyphenyl)propionate and AA using MR methods and whether more clinical studies can prove it as a potential biomarker of AA components. Fructose is an important energy substance in the body, which maintains the normal metabolism of the body, due to the lack of a negative feedback regulation mechanism in its metabolic pathway, it is easy to synthesize fatty acids and gluconeogenesis in the liver. Long‐term large intake of fructose may lead to weight gain, elevated triglyceride levels, and hepatic steatosis, thus inducing cardiovascular diseases, diabetes, and other diseases [68]. The energy metabolism of the human body mainly relies on the conversion between adenosine 5′‐monophosphate (AMP), ADP, and adenosine 5′‐triphosphate (ATP) to maintain the physiological process of the body. ADP is involved in the energy metabolism process of the body and plays a crucial role in maintaining the normal physiological activities of the body, when the ratio of ATP/ADP is reduced, it indicates the lack of energy in the body, therefore, the oxidative phosphorylation rate should be accelerated to maintain the energy supply of the body. It is suggested that ADP to fructose ratio may affect the outcome of AA by affecting the energy metabolism pathway.

In summary, our findings partially align with previous studies; however, there are several limitations to consider in this investigation. Although the study is based on a large sample size and exhibits strong generalizability, it should be noted that the majority of participants were of European descent, necessitating further verification of applicability among other ethnic groups. Although this study establishes a statistical correlation, elucidating the specific biological mechanism requires additional exploration. Future research could validate the association between these metabolites and AA at both cellular and animal model levels. Despite certain limitations in its application, this study serves as an important reference for diagnosing and treating AA.

## 5 Conclusions

This MR study offers valuable insights into the potential causal relationship between blood metabolites and AA risk. The results indicate that disruptions in circulatory metabolic processes may significantly contribute to AA development and progression. Notably, the identification of specific blood metabolites associated with AA risk, such as alpha‐ketoglutarate, propionylcarnitine (C3), xylose, and the ADP to fructose ratio, provides novel pathways for elucidating the disease's underlying mechanisms.

The study highlights the importance of blood‐derived metabolites in precision medicine for AA, enabling early detection of high‐risk individuals, optimizing treatment strategies, and monitoring disease progression. These metabolites serve as biomarkers for screening, prevention, and personalized therapy, supporting pharmacological optimization and real‐time surveillance. Noninvasive detection enhances clinical feasibility, while identified metabolic pathways inform drug development and mechanistic therapies.

Integrating these biomarkers into risk assessment, monitoring, and treatment will advance AA management toward precision medicine, improving outcomes and quality of life. This study confirms the causal relationship between blood metabolites and AA risk and underscores their clinical potential. The findings deepen our understanding of the interplay between genetics, metabolism, and autoimmunity in AA, advancing the field and guiding future research.

## Author Contributions

Conceptualization, data curation, formal analysis, and methodology were conducted by Yi Qu. Funding acquisition, investigation, project administration, resources management, and software were carried out by Jiaxi Huang. Supervision, validation, visualization, writing – original draft, and writing – review and editing were done by Xiaoli Lei.

## Ethics Statement

The ethical considerations are unnecessary as the data are sourced from the “Genomic atlas of the plasma metabolome prioritizes metabolites implicated in human diseases.”

## Conflicts of Interest

The authors declare no conflicts of interest.

## Supporting information


Appendix S1

## Data Availability

The GWAS database (https://www.ebi.ac.uk/gwas/) contains a comprehensive collection of 1400 plasma metabolites. For the European GWAS, the accession number is GCST90199621‐90201020, whereas for the non‐European GWAS it is CST90201021‐90204063. Relevant data on AA can be accessed in the Finngen databases (https://finngen.gitbook.io/documentation/).
